# Intrinsically disordered proteins identified in the aggregate proteome serve as biomarkers of neurodegeneration

**DOI:** 10.1007/s11011-021-00791-8

**Published:** 2021-08-04

**Authors:** Srinivas Ayyadevara, Akshatha Ganne, Meenakshisundaram Balasubramaniam, Robert J. Shmookler Reis

**Affiliations:** 1 Central Arkansas Veterans Healthcare Service, Little Rock, AR 72205 USA; 2 Reynolds Institute on Aging, Department of Geriatrics, University of Arkansas for Medical Sciences, 629 Jack Stephens Drive, Little Rock, AR 72205 USA; 3 BioInformatics Program, University of Arkansas at Little Rock and University of Arkansas for Medical Sciences, Little Rock, AR 72205 USA

**Keywords:** Intrinsically disordered proteins, Protein aggregation, Aggregate proteome, Biomarker, Neurodegneration

## Abstract

A protein’s structure is determined by its amino acid sequence and post-translational modifications, and provides the basis for its physiological functions. Across all organisms, roughly a third of the proteome comprises proteins that contain highly unstructured or intrinsically disordered regions. Proteins comprising or containing extensive unstructured regions are referred to as intrinsically disordered proteins (IDPs). IDPs are believed to participate in complex physiological processes through refolding of IDP regions, dependent on their binding to a diverse array of potential protein partners. They thus play critical roles in the assembly and function of protein complexes. Recent advances in experimental and computational analyses predicted multiple interacting partners for the disordered regions of proteins, implying critical roles in signal transduction and regulation of biological processes. Numerous disordered proteins are sequestered into aggregates in neurodegenerative diseases such as Alzheimer’s disease (AD) where they are enriched even in serum, making them good candidates for serum biomarkers to enable early detection of AD.

## Introduction

Neurodegenerative diseases including Alzheimer’s (AD) and Parkinson’s (PD) diseases are especially prominent as causes of morbidity and mortality in the elderly population (Akushevich et al. 2013). Known risk factors for neurodegenerative diseases are increasing age, oxidative stress, inflammation, hypertension, genetic polymorphism, food habits, depression, and environmental factors (Bertram and Tanzi 2005). Neurodegeneration usually results from a cascade of events believed to involve misfolding of proteins, post-translational modifications, and/or aberrant protein-protein interactions leading to protein aggregation (Ashraf et al. 2014). Proteostasis, i.e., protein homeostasis, depends on the balance between synthesis, proper folding and attainment of an energy efficient structure, as well as appropriate activation of clearance mechanisms for misfolded and aggregated proteins. Several neurological diseases including AD, PD, Huntington’s disease (HD), and Amyotrophic Lateral Sclerosis (ALS), are considered to be protein misfolding disorders that are initiated or characterized by protein misfolding and accumulation into fibrillar or more complex aggregated structures (Ashraf et al. 2014). Familial inheritance of neurodegenerative diseases is often attributable to mutations that destabilize protein structures so as to favor their aggregation. Such mutations direct researchers’ attention to proteins that are crucial in neuropathogenesis (Bertram and Tanzi 2005). In AD, for example, amyloid precursor protein (APP) is cleaved to release Aβ_1–42_ peptide which accumulates as amyloid foci in affected brain regions; familial-AD mutations in APP, such as APP_Sw_, strongly predispose to the development of amyloid foci and AD dementia (Balasubramaniam et al. 2019). Autosomal-dominant mutations in at least 3 genes (*APP, PSEN1* and *PSEN2*) have been shown to be causal for familial AD (Walter et al. 2018). Molecular changes, exacerbated by aging and eventually leading to cognitive loss, are among the diagnostic characteristics of AD. Despite extensive research to understand proteinopathies, effective drug targets remain elusive. Insoluble aggregates characteristic of specific neurodegenerative diseases include, in addition to their diagnostic “seed” proteins, a variety of misfolded and disordered proteins that are largely shared across several diseases (Ayyadevara et al. 2016). Interestingly, not all disordered proteins have equal propensity to enter aggregates. Proteomic analyses of aggregates from diverse sources have repeatedly identified the same subset of disordered proteins (Ayyadevara et al. 2016), suggesting that specific disordered proteins may help to drive aggregate progression. Knockdown of co-aggregating disordered proteins in AD model systems showed significant protection against aggregation and its associated phenotypic changes (Ayyadevara et al. 2015, 2016, Balasubramaniam et al. 2018, 2019), demonstrating their functional roles in aggregation and their potential as novel therapeutic targets for prevention or therapy of neurodegenerative diseases.

### Disordered proteins in aggregates

Recent studies have shown that a protein can adopt multiple secondary, tertiary and quaternary structures while still retaining full functionality (Dill et al. 2008). Although X-ray crystallography has been instrumental in understanding protein structure, proteins should not be regarded as rigid crystal-like entities; even the most stably-folded structure will have some degree of conformational flexibility, and alternative protein structures often interconvert dynamically. Even modest perturbations, such as thermal fluctuations, can alter the lowest-energy protein structure. Several alternative structures that a protein can adopt included structured (folded), molten globular, pre-molten globular, and unstructured (Deiana et al. 2019). Both structured and unstructured proteins tend to misfold as a consequence of oxidative stress or excessive PTM, causing structural distortions that may lead to aggregation and may be associated with pathogenesis of neurodegenerative diseases such as AD, PD, and ALS (Zerovnik et al. 2020).

Many protein structures entered into the protein data bank PDB are not consistently or rigidly maintained across time and diverse conditions. Moreover, many protein structures have missing regions due to the failure to capture a unique x-ray diffraction pattern in that particular area. X-ray diffraction cannot provide reliable information about intrinsically disordered protein regions due to their high propensity for protein-structure fluctuations (Deiana et al. 2019). In contrast, nuclear magnetic resonance (NMR) has provided a more complete characterization of many partially-unstructured proteins that were later classified as intrinsically disordered proteins (IDPs) or as possessing IDP regions. IDPs and IDP regions are flexible due to highly dynamic segments, characterized by complete or nearly complete absence of ordered structure under physiological conditions (Theillet et al. 2016).

A variety of names have been applied in the literature to describe IDPs, including unfolded, unstructured, or denatured protein states. Computational studies have helped to characterize IDPs based on their 3-dimensional structures and atomistic “behavior” in molecular-dynamic simulations. IDPs differ from other globular protein structures in many aspects of their amino acid sequence. Computational studies carried out during the 1990’s showed that IDPs and IDP regions have amino acid sequences significantly different from those of structured proteins. Some pertinent characteristics are the low sequence complexity of IDPs relative to folded proteins, their low content of aromatic and other hydrophobic residues, and increased levels of proline (which disrupts alpha helices) (Chen et al. 2020). In view of their disorder, IDPs also acquire (tolerate) amino acid substitutions at a relatively high rate over evolutionary time. Common physiological deviations, such as changes in intracellular environmental characteristics (pH, ionic strength), as well as PTMs, alter the structural stability of proteins (Fonin et al. 2019). Because disordered proteins are very flexible, IDP’s play important roles in cellular-transition processes such as the regulation of transcription, translation, cell cycle progression, and diverse signaling pathways.

### Intrinsically disordered proteins (IDP)

Diverse neurodegenerative diseases, affecting a variety of pathological and biochemical pathways, are characterized by disease-specific accumulation of aggregates containing many intrinsically disordered proteins (IDPs). A characteristic feature of IDPs is that the same polypeptide takes different conformations, with different consequences, depending on the presence of ligands, other interacting proteins, posttranslational modifications (PTMs), or elevated temperature (Radivojac et al. 2007). We and other researchers have observed several PTMs impacting IDPs that may also contribute to the etiology of a neurological disease. These changes to IDPs might lead to interaction with multiple protein partners, driving the formation of complex aggregates such as plaques and tangles (Oldfield and Dunker 2014). Increasing evidence implies that changes to the unfolded protein response (UPR) frequently contribute to the development of neurodegenerative diseases. Neurons like other cell types have an extensive protein-repair system (Liu et al. 2013; Parcon et al. 2018), localized chiefly to the endoplasmic reticulum (ER), which helps them detect and refold (or remove) misfolded proteins to prevent neurological damage. This process, known as the ER unfolded protein response (or UPR^ER^), plays a key role in protein homeostasis.

Under conditions of ER stress, which is believed to reflect primarily the cumulative toll of misfolded nascent proteins extruded into the rough ER by attached ribosomes, three parallel and partially independent signaling pathways for unfolded protein response are activated: PERK, IRE1 and ATF6 (Walter et al. 2018). These signaling pathways are distinguished by different transmembrane proteins that act as ER-stress sensors: RNA-activated protein kinase R (PKR)-like ER kinase (PERK), activating transcription factor 6 (ATF6), and inositol requiring enzyme 1 (IRE1) (Walter et al. 2018). PERK phosphorylates eIF2α, which leads to an immediate decrease in protein synthesis and in turn activates ATF-4 which then elevates the transcription of downstream UPR-responsive genes. ATF6 and IRE1 also activate the transcription of *ATF4* and *XBP1* genes, respectively, thereby increasing the cell’s protein-folding capacity and alleviating the protein-misfolding burden in the ER (Walter et al. 2018).

### Functional roles of intrinsically disordered proteins

As IDPs are frequently implicated in the development of diseases, in recent years they have become attractive therapeutic targets. However, the dynamic nature of IDPs makes it challenging to design drugs that specifically target a protein of indeterminate structure (Santofimia-Castano et al. 2020). Some IDPs are become destabilized to favor a state transition, which is accompanied by binding to a new partner. As mentioned earlier, our interactome analysis showed that AD aggregates contain many IDPs that adhere together in aggregates. Knockdown of such IDPs may confer protection against aggregation in one or several neurodegenerative models (Balasubramaniam et al. 2019) emphasizing the versatility and therapeutic value of IDPs as drug targets to treat neurodegenerative diseases. As noted, targeting IDP with small-molecule inhibitors is a challenging task due to their lack of tertiary structure. Therefore we propose a novel, feasible approach to targeting IDPs, by screening for small molecules to disrupt specific protein-protein interfaces identified in the aggregate interactome. In this way, targeted small molecules can be custom designed to block particular protein-protein interactions that contribute strongly to pathologic aggregation.

### Computational prediction of IDPs and their physiological function

A number of sophisticated methods have been used to derive computational predictors for IDPs and their disordered regions (Xue et al. 2010). Various methods based on properties as simple as amino-acid composition have proven to predict IDPs and regions with surprising accuracy. Studies implicating IDPs and IDP regions in a wide range of biological activities peaked between the years 2000–2018 (Radivojac et al. 2007).

Extensive studies have been carried out to learn which features of IDP protein sequences predispose them to aggregation. These studies revealed that low-entropy and low-complexity IDP sequences are enriched in aggregates. Low hydrophobic amino-acid contribution is a critical feature of disordered proteins that predicts aggregation. Predicted disordered proteins have low content of hydrophobic residues compared to the predicted ordered proteins. The number of hydrophobic residues in the ordered regions was shown to be higher when compared to the disordered regions resulting in disfavoring the folding mechanism of the protein (Basu et al. 2017). The frequency of occurrence of dipeptides like SS, PP, PG, RR, AA, LE, AE, EE, and GP was found to be high in predicted IDP regions, and also it was shown that the five order-promoting residues (W, F, Y, I, and M) were seen in very few predicted IDP/IDRs (Saravanan et al. 2018). The secondary structure of an IDP is known to comprise chiefly coiled coils. A number of sophisticated methods can be used to computationally derive predictors for IDPs and intrinsically disordered regions (IDRs) directly from amino acid sequences. PONDR, Foldindex, Globplot, DisEMBL, DISOPRED and DISOPRED2 etc. are a few of the tools used to predict IDPs/IDRs; they can be accessed via publically available servers (Wright and Dyson 2015).

DisProt version 8 is a curated database created for IDPs and IDRs, utilizing 32 IDP-related functional subclasses. The database has been upgraded so as to assign each disordered protein a unique identifier. The number of disordered proteins has been expanded to 1390, plus 3041 IDRs.

Some of the key roles of IDPs are to act as molecular switches, transcriptional activators, entropic bristles, and entropic clocks. Small heat shock proteins (sHSPs), a large family of low-molecular-weight proteins (13–43 kDa) that consist of long intrinsically disordered regions, play important roles in the formation of large sHSP oligomers which acts as chaperones (Uversky 2015). IDPs can also serve as thermosensors or thermoswitches, due to their tendency to transition to different structures as the temperature changes. It appears likely that further work will reveal additional functional roles of IDPs.

### Post-translational modification of intrinsically disordered proteins

As discussed above, IDPs undergo multiple conformational changes, which may be necessitated by their multiple physiological functions (Hu et al. 2014). It is well documented that post-translational modifications (PTMs), especially phosphorylation, generally alter the physicochemical properties of a protein. Interestingly, many of the IDPs we identified by proteomic analyses of AD aggregates were phosphorylated. Some of these phosphorylated IDPs were absent from AMC aggregates (see Supplementary data in (Ayyadevara et al. 2016), suggesting that PTMs (especially phosphorylation) may contribute to AD neuropathology, presumably by increasing the aggregation propensity of IDPs. PTMs can modify the physicochemical properties of IDPs, leading to structural alterations that in turn are expected to affect the normal biological properties of IDPs (e.g., protein-protein interactions), perhaps through exposure of hydrophobic regions that are especially vulnerable to aggregation. One classic example, for illustration, is the sequence of molecular changes mediating aggregation of tau protein. Although under normal conditions, tau interacts with other proteins of the micro-tubule to stabilize its structure, in Alzheimer’s disease it becomes hyperphosphorylated, leading to the formation of neurofibrillary tangles. These intra-neuronal aggregates are a hallmark diagnostic feature of AD, and are believed to reflect the conversion of tau into an IDP through excessive phosphorylation (Ayyadevara et al. 2016).

### IDPs in AD-associated aggregation

We observed a significant enrichment of IDPs in aggregates isolated from hippocampi of AD patients, relative to those from age-matched controls (Ayyadevara et al. 2016). However, not all disordered proteins end up in aggregates, nor is percent disorder alone sufficient to predict protein aggregation. PONDR-FIT (Xue et al. 2010) and Aggrescan (Conchillo-Sole et al. 2007), software designed to predict disorder percentage and aggregation propensity of IDPs respectively in neurodegenerative tissues, identified another 25 properties that distinguish proteins consistently enriched in AD brain aggregates (relative to controls) from those that escape aggregation [A.G. et al. manuscript in preparation].

*C. elegans* orthologs of disordered proteins found in human AD-aggregate proteomics (Balasubramaniam et al. 2019), evaluated for functional roles in nematode models of AD, consistently improved the age-dependent chemotaxis phenotype when knocked down by RNA interference. These orthologs were evaluated by neural networks and found to be influential in alleviating the chemotaxis deficit mediated by leaky amyloid expression in neurons of aged worms (Balasubramaniam et al. 2019). The most influential proteins, based on their enrichment in AD brain aggregates, can be further evaluated to assess their therapeutic potential as drug targets.

**Fig. 1:**
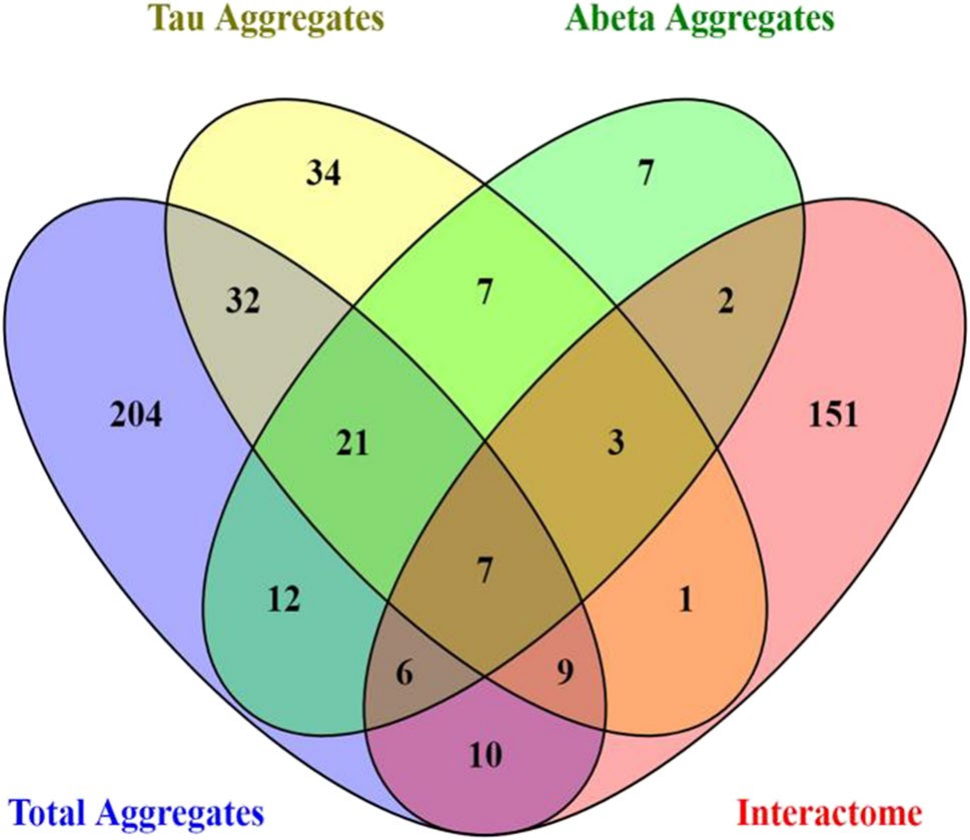
Overlap of IDPs in Alzheimer’s disease insoluble aggregate proteome

In cells, IDPs often operate in extremely crowded environments and rapidly interconvert among alternative conformations. We can classify their final states as either folded, unfolded, or non-foldable. IDPs have a higher propensity to aggregate with aging and/or as a consequence of a disease state, in either case showing enrichment in the aggregate proteome. Figure 1 shows a Venn diagram representing IDPs in four overlapping aggregate proteomes (Ayyadevara et al. 2016; Balasubramaniam et al. 2019). The relative abundances of IDPs in aggregates far exceeds their representation in the human proteome. Most human proteins are not disordered, with normal IDP frequency estimated to be ~33% (Deiana et al. 2019). However, IDPs comprise ~51% of sarkosyl-insoluble proteins identified in Aβ- or tau-specific immunopulldown aggregates, as well as non-pulldown aggregates isolated from either AD or AMC hippocampi (Ayyadevara et al. 2016). The number of spectral hits for aggregate IDPs was also 20% higher in AD hippocampi than in age-matched controls (Ayyadevara et al. 2016). We studied the aggregate interactomes of a cell-culture model of AD: human SY5Y-APP_Sw_ neuroblastoma cells expressing an amyloid-predisposed familial-Alzheimer’s APP mutant termed APPSw. These interactomes comprise 270 hub and hub-connector proteins, of which 189 (70%) are disordered. That analysis of aggregate interactomes implies that IDPs are not only preferentially sequestered into aggregates, but also heavily cross-linked to adjoining proteins. The actual contribution of intrinsically disordered regions to aggregate formation and progression may be even greater, because other proteins in the aggregate proteome may also contain disordered regions that arise due to PTMs or protein: protein interactions. Moreover, IDP-interacting proteins, although not themselves IDPs, may be co-opted into aggregates by those interactions. IDPs in aggregates provide a rich source of non-invasive biomarkers due to their representation in serum. Remarkably, 68 of the 91 proteins in Aβ-specific aggregates (75%), and 81 of the 230 proteins in tau-specific aggregates (35%) are also enriched in the serum proteomes of Alzheimer's disease subjects, relative to controls (Dey et al. 2019).

## Conclusions

With recent advances in the understanding of protein disorder in cell biology, it is now recognized that IDPs or IDP regions (intrinsically disordered regions of proteins) govern a diverse variety of cellular processes. Technical improvements in protein crosslinking, recently applied to protein aggregation studies (Balasubramaniam et al. 2019), hold considerable promise for future studies intended to characterize and understand the roles of IDPs. One of the most exciting developments has been the identification of IDPs and their interacting partners in aggregates found within the cell. While such studies are still relatively new, they hold considerable promise for the identification of novel biomarkers and therapeutic targets in neurodegenerative diseases. A comprehensive understanding of IDPs requires their localization to subcellular compartments, and evaluation of their roles in normal signal transduction, development, aging, and disease. This could lead to their deployment as disease-predictive biomarkers and reporters of protein-unfolding stresses and of subtle changes in the cellular environment.

## Data Availability

There are no new data in this paper; Fig. 1 is a new image summarizing a variety of proteomics data that have been published previously. All data discussed have been published in peer-reviewed journals, for which references are provided. We will respond to any requests from the research community, and will make all original raw data available upon request.
